# MULTISENSORY INTEGRATION IN AGING: LINKS TO MOBILITY

**DOI:** 10.1093/geroni/igae098.1071

**Published:** 2024-12-31

**Authors:** Jeannette Mahoney

**Affiliations:** Stony Brook Medicine, Stony Brook, New York, United States

## Abstract

Multisensory integration, a rapidly growing field of neuroscience, investigates the simultaneous processing of information from multiple sensory systems. Researchers argue that our brains are designed to simultaneously process information from multiple sensory inputs to produce the most appropriate response to environmental cues. Studies in primates and young adults reveal that flexible multisensory integration is regulated by PFC areas, including dorsomedial and ventrolateral subregions. In fact, considerable strides have been made in defining the underlying properties, neural substrates, and timing of multisensory integration effects in healthy young adults; however, multisensory research in older adults has not been studied comprehensively. Further understanding of multisensory integration in aging could prove valuable, given that increased sensory deficits are associated with aging. Age-related visual and somatosensory impairments have been individually linked to slower gait, functional decline, increased risks of falls, and poorer quality of life. In a series of studies, we provide evidence for robust, but differential visual-somatosensory integration (VSI) effects in healthy aging and report its significant link with clinically-meaningful outcomes. Specifically, we show that older adults with intact levels of VSI demonstrate better balance, faster gait velocity and lower incidence of falls, compared to those with poor VSI. Additionally, we reveal that older adults with MCI and dementia demonstrate significantly reduced magnitude of VSI compared to those without cognitive impairments, which in turn was associated with worse unipedal stance and gait performance. Our current research objectives include elucidating whether VSI is a novel, non-invasive, non-cognitive predictor of Alzheimer’s disease.

